# Referral of people with low back pain to physical therapists in Brazilian primary healthcare: A challenge revealed

**DOI:** 10.1016/j.bjpt.2025.101538

**Published:** 2025-09-30

**Authors:** Tais Luciana Lacerda, Pedro Lacerda Montes, Luciana Gazzi Macedo, Raymond Ostelo, Henry Maia Peixoto, Rodrigo Luiz Carregaro

**Affiliations:** a Universidade de Brasília (UnB), Núcleo de Evidências e Tecnologias em Saúde (NETecS), Brasília, Brazil; b McMaster University, School of Rehabilitation Science, Hamilton, Canada; c Master Program in Rehabilitation Sciences (PPGCR), Universidade de Brasília (UnB), Brasília, Brazil; d Department of Health Sciences, Faculty of Science, VU University Amsterdam, Amsterdam Movement Sciences research institute Amsterdam, Amsterdam, The Netherlands; e Department of Epidemiology and Data Science, Amsterdam University Medical Centre, Location Vrije Universiteit, Amsterdam, The Netherlands

**Keywords:** Back pain, Primary health care, Prevention, Public health

## Abstract

**Background:**

Low back pain (LBP) is a disabling condition worldwide, and current evidence suggests low rates of referral to physical therapists and extensive use of low-value interventions such as pharmacological treatments and emergency visits.

**Objective:**

To investigate the frequency of referrals and characterize people with LBP accessing primary care physical therapists, as well as characterize clinical and sociodemographic aspects and the use of health resources in Brazil.

**Method:**

Observational study using nationwide data on 1,459,710 adults with LBP, stratified according to G1: medical care only, G2: medical care and referral to physical therapist, G3: physical therapist as first contact. Data were analyzed descriptively.

**Results:**

1,405,145 people with LBP were included in G1, followed by G2 (N:14,079), and G3 (N:40,486). The majority was female (56.3 %), and the average age was 49 (±17) years for females and 48 (±17) for males. Less than 1 % (G2) were referred to physical therapists. Of these, 8085 (57.4 %) had an average duration of 17.4 days (±65.6) between referral and their clinical appointment, and 5994 (42.6 %) had a longer duration (261.1 ± 146.9 days). A total of 130,570 (8.9 %) participants were referred for imaging, totaling 152,150 exams. G1 had 105.65 exams/1000 people and 128 referrals to specialists/1000 people. G2 had 196.32 exams and 384.76 referrals to specialists/1000 people, and G3 had 22.87 exams and 64.89 referrals to specialists/1000 people.

**Conclusion:**

We found a relatively low number of referrals of people with LBP to physical therapists in primary health care in Brazil, and a long period between the referral and the first contact with this professional. In addition, diagnostic imaging and referral to a specialist were frequently used.

## Highlights

• From a national primary care perspective, around 1 % of people with LBP were referred to physical therapy.
• The amount of time between referral and being seen by physical therapists ranged from 17 to 261 days.
• The overall rate of physical therapy visits to manage LBP was 1.28 per 1000 people.

## Introduction

Low back pain (LBP) is a global public health problem characterized by high prevalence and high rates of disability^1^ leading to productivity losses.^2^ In Brazil, Primary Health Care (PHC) is the gateway to the public health system, where nearly 85 % of the population's health needs must be met.^3^ In addition, LBP is the most common musculoskeletal condition treated by physical therapists in PHC settings.^4^^,^^5^

Early access to physical therapy for individuals with LBP is internationally recommended^6^, ^7^, ^8^, ^9^ because it is deemed to be cost-effective and has been found to significantly reduce disability and the use of low-value health resources.^7^^,^^9^, ^10^, ^11^ Recent evidence has identified that when physical therapists are the first point of contact within PHC, for musculoskeletal disorders, there is improved quality of life, reduced pain, and lower risk of developing chronic pain.^7^^,^^10^^,^^12^^,^^13^ Current guidelines suggest the use of non-pharmacological treatments, such as health education and exercise therapies.^14^, ^15^, ^16^ Furthermore, guidelines discourage the routine use of imaging tests, especially for non-specific LBP.^16^^,^^17^ Such evidence supports the Brazilian Ministry of Health's recommendations,^18^ towards the use of health education and counselling, as well as regular exercise, and discourages unnecessary medicalization and referral for imaging tests in the absence of red flags. In Brazil, since 2008, physical therapists have been present in PHCs within Family Health Support Centers (NASF-AB, currently known as Multidisciplinary Teams or e-Multi), an integrated multiprofessional team linked to Family Health Teams (FHT). Access to e-Multi professionals, including physical therapists, must be by referral and not as a first access professional.^19^ Accordingly, physical therapy interventions are aimed at health promotion, prevention, and rehabilitation (e.g., exercise therapy, health education).^14^^,^^15^^,^^19^

The Ministry of Health recommends that people with LBP be followed up through PHC,^3^^,^^20^ with referral to specialists only when clinical treatment has failed or when there are red flags.^20^^,^^21^ However, previous studies in Brazil have shown that the service most used by this population is the emergency department, and one of the most common treatments is pharmacological.^22^^,^^23^ Other relevant aspect is the low rate of referral to physical therapy,^22^ and the low implementation of guidelines recommendations.^24^ This context is reflected internationally, as despite existing guidelines, the management of LBP within PHC is not common.^25^

Although physical therapy interventions are recommended by clinical guidelines, a previous study have shown conflicting evidence due to the low number of referrals to this profession.^22^ Therefore, considering that referral to physical therapy was associated with lower healthcare costs,^7^^,^^9^^,^^11^^,^^26^ we designed this study to investigate the frequency of referrals of people with LBP to physical therapists in primary care from a national perspective using real-world data from the public health system (i.e., e-SUS). We also aimed to characterize clinical and sociodemographic aspects and health care resource use of people with LBP, and to estimate the quantity, frequency, and timing of referrals to physical therapy.

## Method

### Study design

This is an observational study using primary care data from the Public Health System Database (e-SUS), obtained through the Health Database for Primary Care (SIS-AB). Data were extracted from the Individual Health Care File (FAI) and the Individual Registration File (FCI) used by the health system. They were provided anonymously in the form of a list of data organized by date. All states in the country were included.

### Health condition and target population

Data from 1,615,508 individuals were analyzed. Individuals with complaints of low back pain who were treated at a PHC and registered in an electronic medical record (e-SUS) between 1 January 2019 and 31 December 2020 were eligible for inclusion. The selection period of participants considered the implementation of the e-SUS system, which took place in 2018, and the start of the COVID-19 pandemic, which caused major changes in the work routines after 2020.

The following codes from (a) the International Classification of Diseases - 10th edition (ICD-10) and (b) the International Classification of Primary Care - 2nd edition (CIAP-2) related to low back pain were used to define the eligible population: (a) M25.7, M51, M51.1, M51.9, M53.8, M53.9, M54, M54.1, M54.3, M54.4, M54.5, M54.6, M54.8, M54.9, M40.4, M40.5, M41, M41.2, M41.9, M42.9, M48, M 48.0, M48.9; (b) L02, L03, L18, L85, L86.

Records were organized by individual (anonymized) in chronological order and stratified by state and region. Duplicate records were excluded from analysis. To characterize the referrals, the included participants were divided into groups using as criteria data from the first consultation related to LBP and performed by a physician or physical therapist. Nurses and allied health professionals were not included for group classification because there was not enough data to identify the type of consultation or procedure.

To meet the inclusion and grouping criteria, we used the category of professional providing the service (doctor or physical therapist), the date and location of the health service, the referral to physical therapy, specialist and imaging tests, and the time of referral. Participants were thus stratified into three groups: G1) consultation with a physician only, without referral or assistance from a physical therapist; G2) consultation with a physician and referral to a physical therapist; G3) consultation with a physical therapist as first contact.

We defined a referral to physical therapy care in the PHC (G2) when a patient had a consultation with a doctor and a subsequent consultation with a physical therapist for LBP. The time of referral was then defined by the number of days between the first medical appointment and the first physical therapy appointment.

### Variables and definitions

Studies on the prevalence of LBP in Brazil showed associations between gender, lifestyle habits and biopsychosocial conditions, and the sociodemographic profile.^27^^,^^28^ However, such variables were not available in the dataset analyzed. Given this gap, we decided to use age, sex, and education as proxies to represent the sociodemographic profile of the target population.

We analyzed the following components of healthcare: medical care, physical therapy care, referral to a specialist, and the amount of imaging used to determine whether clinical guideline recommendations were followed. There were no records of other health services, such as drugs or other procedures.

Although previous studies have considered different periods to define early or late referral, ranging from 3 to 90 days^6^^,^^8^^,^^10^^,^^11^^,^^25^^,^^29^^,^^30^ we adopted early, and late referral classifications based on Brazilian regulations used by the public health system. This regulation considers the need for priority care for health conditions where the referral or initial diagnosis occurred within 90 days.^21^ The LCLP states that in cases where there is no need for care at the secondary level, the individual should be referred to physical therapy and a multidisciplinary team at the initial consultation.^4^ Therefore, 90 days was considered early referral, and this period was used to examine the timing of referral and similarity with previous studies.

### Data analysis

Data were analyzed using the Pandas library in Python and Microsoft Excel.

As we analyzed all records - not a sample - from primary care systems (i.e., Public Health System Database - e-SUS, and the Health Database for Primary Care - SIS-AB), there is no expectation of random error that would justify the use of any statistical inference technique. Thus, descriptive statistics were performed using means (standard deviations), medians (quartiles), or frequencies (percentages), to characterize the referrals for each group and category. Measures describing population characteristics in terms of gender, age, and education were calculated based on the total number of patients included in the study. Measures related to the proportion of medical and physical therapy consultations, referrals to specialists, and requests for tests were calculated using the population of each Brazilian state as the denominator, to describe the use of health resources in each state.

## Results

Data from 1,615,508 individuals were analyzed for eligibility. Of these, 155,798 did not meet the inclusion criteria because their first consultation was with another professional. A total of 1,459,710 participants were included in the groups as follows: G1) 1,405,145 individuals, G2) 14,079 individuals and G3) 40,486 individuals.

The characteristics of the participants included are shown in Table 1. Most of the individuals were female 56.3 % (N: 909,303). The mean age was 49 (± 17) years for women and 48 (± 17) years for men. The most common level of education was primary school (21.5 %; N: 346,570), followed by secondary school (20.2 %; N: 325,602).

**Table 1:** Characteristics of the included participants.

Region	State	Sex - n ( %)		Age (years; Mean, SD)		Educational Level - n ( %)					
		F	M	F	M		P1	P2	S	T	Other
North	AC	1489 (0.09 %)	701 (0.04 %)	43 ± 16	45 ± 17		589 (0.04 %)	421 (0.03 %)	365 (0.02 %)	95 (0.01 %)	207 (0.01 %)
	AM	19,426 (1.20 %)	11,619 (0.72 %)	44 ± 16	45 ± 17		5427 (0.34 %)	8047 (0.50 %)	8387 (0.52 %)	1727 (0.11 %)	1448 (0.09 %)
	AP	0 (0.00 %)	4 (0.00 %)	–	30 ± 8		0 (0.00 %)	1 (0.00 %)	2 (0.00 %)	1 (0.00 %)	0 (0.00 %)
	PA	13,466 (0.83 %)	7218 (0.45 %)	44 ± 17	45 ± 18		4997 (0.31 %)	5120 (0.32 %)	3982 (0.25 %)	845 (0.05 %)	1311 (0.08 %)
	RO	18,303 (1.13 %)	10,519 (0.01 %)	46 ± 16	47 ± 17		7189 (0.44 %)	6438 (0.40 %)	4768 (0.30 %)	1336 (0.08 %)	1463 (0.09 %)
	RR	139 (0.01 %)	68 (0.00 %)	43 ± 17	49 ± 18		28 (0.00 %)	48 (0.00 %)	81 (0.01 %)	17 (0.00 %)	7 (0.00 %)
	TO	29,118 (1.80 %)	18,433 (1.14 %)	46 ± 17	47 ± 18		11,410 (0.71 %)	11,480 (0.71 %)	10,937 (0.68 %)	3663 (0.23 %)	3103 (0.19 %)
*SUBTOTAL*		*81,941* *(5.07 %)*	*48,562 (3.01 %)*	*45±16*	*46±17*		*29,640* *(1.83 %)*	*31,555* *(1.95 %)*	*28,522* *(1.77 %)*	*7684* *(0.48 %)*	*7539* *(0.47 %)*
Northeast	AL	11,444 (0.71 %)	6018 (0.37 %)	47 ± 16	46 ± 16		4638 (0.29 %)	3574 (0.22 %)	2499 (0.15 %)	667 (0.04 %)	2172 (0.13 %)
	BA	57,557 (3.56 %)	27,692 (1.71 %)	49 ± 17	49 ± 18		28,375 (1.76 %)	18,376 (1.14 %)	11,955 (0.74 %)	2158 (0.13 %)	8863 (0.55 %)
	CE	23,568 (1.46 %)	11,440 (0.71 %)	46 ± 17	45 ± 18		9935 (0.61 %)	8203 (0.51 %)	7279 (0.45 %)	1731 (0.11 %)	2992 (0.19 %)
	MA	12,603 (0.78 %)	5743 (0.36 %)	45 ± 17	46 ± 18		4443 (0.28 %)	4558 (0.28 %)	4425 (0.27 %)	964 (0.06 %)	1459 (0.09 %)
	PB	30,227 (1.87 %)	15,366 (0.95 %)	48 ± 17	47 ± 18		15,069 (0.93 %)	10,369 (0.64 %)	7702 (0.48 %)	2015 (0.12 %)	4138 (0.26 %)
	PE	37,453 (2.32 %)	17,263 (1.07 %)	47 ± 17	46 ± 17		12,575 (0.78 %)	12,814 (0.79 %)	9196 (0.57 %)	1611 (0.10 %)	4174 (0.26 %)
	PI	26,456 (1.64 %)	14,686 (0.91 %)	46 ± 17	46 ± 18		11,011 (0.68 %)	8091 (0.50 %)	7744 (0.48 %)	2104 (0.13 %)	3323 (0.21 %)
	RN	16,681 (1.03 %)	8997 (0.56 %)	47 ± 17	47 ± 17		7259 (0.45 %)	6649 (0.41 %)	4837 (0.30 %)	1191 (0.07 %)	1868 (0.12 %)
	SE	1312 (0.08 %)	496 (0.03 %)	48 ± 16	46 ± 16		467 (0.03 %)	381 (0.02 %)	263 (0.02 %)	57 (0.00 %)	132 (0.01 %)
*SUBTOTAL*		*217,301* *(13.45 %)*	*107,701* *(6.67 %)*	*47±17*	*47±18*		*93,772* *(5.80 %)*	*73,015* *(4.52 %)*	*55,900* *(3.46 %)*	*12,498* *0.77 %)*	*29,121* *(1.80 %)*
Central West	DF	22,614 (1.40 %)	10,613 (0.66 %)	46 ± 16	46 ± 17		4128 (0.26 %)	6570 (0.41 %)	7658 (0.47 %)	2143 (0.13 %)	981 (0.06 %)
	GO	34,280 (2.12 %)	20,153 (1.25 %)	49 ± 16	49 ± 17		13,987 (0.87 %)	14,218 (0.88 %)	8601 (0.53 %)	2353 (0.15 %)	2906 (0.18 %)
	MS	30,196 (1.87 %)	17,660 (1.09 %)	49 ± 16	47 ± 18		7139 (0.44 %)	10,875 (0.67 %)	6508 (0.40 %)	1641 (0.10 %)	2539 (0.16 %)
	MT	34,828 (2.16 %)	21,277 (1.32 %)	46 ± 16	46 ± 17		10,883 (0.67 %)	13,567 (0.84 %)	9429 (0.58 %)	3035 (0.19 %)	2593 (0.16 %)
*SUBTOTAL*		*121,918* *(7.55 %)*	*69,703* *(4.31 %)*	*48±16*	*47±18*		*36,137* *(2.24 %)*	*45,230* *(2.80 %)*	*32,196* *(1.99 %)*	*9172* *(0.57 %)*	*9019* *(0.56 %)*
Southeast	ES	4534 (0.28 %)	2257 (0.14 %)	50 ± 16	48 ± 18		1449 (0.09 %)	1380 (0.09 %)	1084 (0.07 %)	285 (0.02 %)	383 (0.02 %)
	MG	121,501 (7.52 %)	72,625 (4.50 %)	50 ± 17	48 ± 18		51,891 (3.21 %)	43,596 (2.70 %)	31,838 (1.97 %)	6944 (0.43 %)	10,021 (0.62 %)
	RJ	62,400 (3.86 %)	29,009 (1.80 %)	50 ± 17	48 ± 17		22,358 (1.38 %)	20,014 (1.24 %)	21,321 (1.32 %)	2362 (0.15 %)	1719 (0.11 %)
	SP	117,236 (7.26 %)	66,223 (4.10 %)	50 ± 17	48 ± 17		36,346 (2.25 %)	39,774 (2.46 %)	35,787 (2.22 %)	6872 (0.43 %)	6168 (0.38 %)
*SUBTOTAL*		*305,671* *(18.92 %)*	*170,114* *(10.53 %)*	*50±17*	*48±17*		*112,044* *(6.94 %)*	*104,764* *(6.48 %)*	*90,030* *(5.57 %)*	*16,463* *(1.02 %)*	*18,291* *(1.13 %)*
South	PR	29,188 (1.81 %)	16,649 (1.03 %)	50 ± 17	48 ± 18		11,009 (0.68 %)	8671 (0.54 %)	6380 (0.39 %)	1449 (0.09 %)	2436 (0.15 %)
	RS	99,580 (6.16 %)	60,017 (3.72 %)	51 ± 17	49 ± 17		37,373 (2.31 %)	42,512 (2.63 %)	19,843 (1.23 %)	4757 (0.29 %)	6410 (0.40 %)
	SC	53,704 (3.32 %)	35,858 (2.22 %)	49 ± 17	47 ± 17		26,595 (1.65 %)	19,855 (1.23 %)	14,369 (0.89 %)	4358 (0.27 %)	3114 (0.19 %)
*SUBTOTAL*		*182,472* *(11.30 %)*	*112,524* *(6.97 %)*	*50±17*	*48±17*		*74,977* *(4.64 %)*	*71,038* *(4.40 %)*	*40,592* *(2.51 %)*	*10,564* *(0.65 %)*	*11,960* *(0.74 %)*
**TOTAL**		**909,303** **(56.29 %)**	**508,604** **(31.48 %)**	**49±17**	**48±17**		**346,570** **(21.45 %)**	**325,602** **(20.15 %)**	**247,240** **(15.30 %)**	**56,381** **(3.49 %)**	**75,930** **(4.70 %)**

Elementary Education is divided into: Elementary School 1 (1st to 4th school years) and Elementary School 2 (5th to 9th years).

⁘Number of individuals with educational level left in blank or unknown = 563,785 (34.90 %).

◌Number of individuals with sex field left blank or unknown = 197,601 (12.23 %).

Total per region: % relation to total sample (Brazil).

Missing data: education = 563,785 individuals (34.90 %); sex = 197,601 individuals (12.23 %).

F: female; M: male; P1: Elementary education 1; P2: Elementary education 2; S: Secondary education (High School); T: Tertiary education (College);.

States: AC - Acre; AM - Amazonas; AP - Amapá; PA - Pará; RO - Rondônia; RR - Roraima; TO - Tocantins, AL - Alagoas; BA - Bahia; CE - Ceará; RJ - Rio de Janeiro; SP - São Paulo; PR - Paraná; RS - Rio Grande do Sul; SC - Santa Catarina; MA - Maranhão; PB - Paraíba; PE - Pernambuco; PI - Piauí; RN - Rio Grande do Norte; SE - Sergipe; DF - Distrito Federal; GO - Goiás; MS - Mato Grosso do Sul; MT - Mato Grosso; ES - Espírito Santo, MG - Minas Gerais.

Data on the proportion of medical and physical therapy consultations in the regions and states of Brazil are shown in Table 2.

**Table 2:** Rate of medical and physical therapy consultations (n/1000 people), according to region and state. Brazil 2019–2020.

Region	State	Medical consultations	Physical therapy consultations
	AC	3.40	<0.01
	AM	10.99	0.87
	AP	<0.01	<0.01
North	PA	3.11	0.26
	RO	24.70	1.43
	RR	0.50	0.01
	TO	38.44	11.61
*SUBTOTAL*		*9.77*	*1.44*
	AL	6.35	0.39
	BA	7.60	0.44
	CE	3.95	0.37
	MA	3.16	0.13
Northeast	PB	13.53	1.50
	PE	6.92	0.51
	PI	14.93	3.70
	RN	9.10	1.65
	SE	0.76	0.11
*SUBTOTAL*		*6.93*	*0.72*
	DF	14.09	1.38
Central-West	GO	11.40	1.37
	MS	23.90	2.62
	MT	22.82	0.41
*SUBTOTAL*		*16.47*	*1.38*
	ES	2.09	0.17
Southeast	MG	11.21	1.42
	RJ	6.47	0.53
	SP	5.96	1.02
*SUBTOTAL*		*7.14*	*0.98*
	PR	5.64	1.45
South	RS	21.04	3.44
	SC	18.15	4.93
*SUBTOTAL*	*14.47*	*3.04*	
**TOTAL**	**9.08**	**1.28**	

States: AC - Acre; AM - Amazonas; AP - Amapá; PA - Pará; RO - Rondônia; RR - Roraima; TO - Tocantins, AL - Alagoas; BA - Bahia; CE - Ceará; RJ - Rio de Janeiro; SP - São Paulo; PR - Paraná; RS - Rio Grande do Sul; SC - Santa Catarina; MA - Maranhão; PB - Paraíba; PE - Pernambuco; PI - Piauí; RN - Rio Grande do Norte; SE - Sergipe; DF - Distrito Federal; GO - Goiás; MS - Mato Grosso do Sul; MT - Mato Grosso; ES - Espírito Santo, MG - Minas Gerais.

We found a total of 2,177,086 medical or physical therapy consultations for LBP between 2019 and 2020. There were about 9.08 medical consultations per 1000 people in Brazil. The region with the highest rate of medical consultations was the Central West (16.5/1000). Tocantins was the state with the highest rate of this service (38.4/1000).

The rate of physical therapy visits in Brazil was 1.28 per 1000 inhabitants. The South region had the highest rate (3.4/1000) among the other regions, and Tocantins had the highest rate of physical therapy visits (11.61/1000).

We found that less than 1 % (G2; N: 14,079) of participants with LBP complaints were referred to a physical therapist. Of these, 8085 (57.4 %) had an average early referral duration of 17.4 days (± 65.6) and 5994 participants (42.6 %) had a late referral (261.1 ± 146.9 days).

Table 3 provides detailed information on the healthcare resources used. A total of 130,570 (8.9 %) participants were referred for imaging, for a total of 152,150 examinations. The South region had the highest referral rate (1.23 tests/1000). A total of 187,939 people (12.9 %) were referred to a specialist, with the Central-West region having the highest rate of specialist referrals.

**Table 3:** Rate of diagnostic imaging use and referral to specialists and physical therapists (n/1000 people), according to region and state. Brazil 2019–2020.

Region	State	Imaging Test	Referral to Medical Specialist	Referral to Physical Therapist
North	AC	0.21	0.33	0.00
	AM	1.48	1.23	0.05
	AP	<0.01	<0.01	<0.01
	PA	0.23	0.25	0.02
	RO	2.01	3.32	0.07
	RR	<0.01	0.07	<0.01
	TO	1.99	2.35	0.65
*SUBTOTAL*		*0.82*	*0.93*	*0.08*
Northeast	AL	0.87	0.98	<0.01
	BA	0.39	0.55	0.02
	CE	0.22	0.30	0.02
	MA	0.11	0.10	0.01
	PB	0.76	1.20	0.07
	PE	0.33	0.68	0.03
	PI	1.10	1.55	0.10
	RN	0.63	1.07	0.06
	SE	0.11	0.08	0.00
*SUBTOTAL*		*0.42*	*0.62*	*0.03*
Central-West	DF	0.80	0.79	0.19
	GO	0.84	1.04	0.05
	MS	1.44	1.78	0.16
	MT	1.38	2.06	0.01
*SUBTOTAL*		*1.05*	*1.34*	*0.09*
	ES	0.32	0.36	<0.01
Southeast	MG	0.70	1.05	0.07
	RJ	0.76	0.76	0.04
	SP	0.66	0.99	0.05
*SUBTOTAL*		*0.67*	*0.93*	*0.05*
South	PR	0.38	0.43	0.09
	RS	1.42	1.03	0.18
	SC	2.29	2.07	0.28
*SUBTOTAL*		*1.23*	*1.05*	*0.17*
**TOTAL**		**0.72**	**0.89**	**0.07**

States = AC - Acre; AM - Amazonas; AP - Amapá; PA - Pará; RO - Rondônia; RR - Roraima; TO - Tocantins, AL - Alagoas; BA - Bahia; CE - Ceará; RJ - Rio de Janeiro; SP - São Paulo; PR - Paraná; RS - Rio Grande do Sul; SC - Santa Catarina; MA - Maranhão; PB - Paraíba; PE - Pernambuco; PI - Piauí; RN - Rio Grande do Norte; SE - Sergipe; DF - Distrito Federal; GO - Goiás; MS - Mato Grosso do Sul; MT - Mato Grosso; ES - Espírito Santo, MG - Minas Gerais.

Data on the use of health care resources in each group is shown in Table 4. In G1, there were 105.65 imaging tests and 128.03 specialist referrals per 1000 population. In G2 there were 196.32 imaging tests and 384.76 referrals to specialists per 1000 people, and in G3 there were 22.87 imaging tests and 64.89 referrals to specialists per 1000 people.

**Table 4:** Data on the rate of health services use (n/1000 people), considering referrals group (G1: Consultation only a medical doctor - N: 1405,145); G2: Consultation with a doctor and physical therapist - N: 14,079), and G3: physical therapist as first-contact - N: 40,486), according to region and state. Brazil 2019–2020.

**Region**	**State**	Number of individuals - n ( %)			Health services use					
		G1	G2	G3	Imaging Tests			Medical Specialist consultation		
					G1	G2	G3	G1	G2	G3
North	AC	2413 (0.17 %)	1 (<0.001 %)	0 (<0.001 %)	75.84	<0.001	–	119.35	1000.00	–
	AM	37,152 (2.55 %)	200 (0.01 %)	957 (0.07 %)	163.98	220.00	10.45	135.69	240.00	10.45
	AP	1 (0.00 %)	0 (<0.001 %)	0 (<0.001 %)	<0.001	–	–	<0.001	–	–
	PA	22,271 (1.53 %)	143 (0.01 %)	394 (0.03 %)	88.23	139.86	15.23	94.47	335.66	50.76
	RO	33,750 (2.31 %)	128 (0.01 %)	459 (0.01 %)	105.27	117.19	13.07	170.52	710.94	117.65
	RR	277 (0.02 %)	0 (<0.001 %)	2 (<0.001 %)	3.61	–	<0.001	148.01	–	<0.001
	TO	43,303 (2.97 %)	1021 (0.07 %)	3011 (0.21 %)	69.07	90.11	14.95	76.74	253.67	40.19
*SUBTOTAL*		*139,167* *(9.53 %)*	*1493* *(0.10 %)*	*4823* *(0.33 %)*	*106.24*	*114.53*	*13.89*	*118.94*	*299.40*	*42.50*
Northeast	AL	16,776 (1.15 %)	12 (<0.001)	121 (0.01 %)	172.81	83.33	8.26	195.46	83.33	<0.001
	BA	89,693 (6.14 %)	253 (0.02 %)	1084 (0.07 %)	64.22	142.29	10.15	89.80	308.30	31.37
	CE	28,355 (1.94 %)	15,960 (0.01 %)	575 (0.04 %)	69.86	81.25	6.96	96.31	137.50	31.30
	MA	18,828 (1.29 %)	39 (0.00 %)	196 (0.01 %)	41.85	0.00	0.00	37.71	76.92	10.20
	PB	42,339 (2.90 %)	297 (0.02 %)	969 (0.07 %)	70.81	101.01	6.19	110.23	400.67	54.70
	PE	53,413 (3.66 %)	257 (0.02 %)	1579 (0.11 %)	58.67	163.42	8.23	120.08	303.50	21.53
	PI	39,386 (2.70 %)	322 (0.02 %)	1439 (0.10 %)	90.84	43.48	6.25	126.67	152.17	24.32
	RN	24,869 (1.70 %)	218 (0.01 %)	1031 (0.07 %)	86.77	169.72	10.67	146.29	348.62	45.59
	SE	1486 (0.10 %)	4 (0.00 %)	51 (0.00 %)	172.95	0.00	0.00	127.19	0.00	0.00
*SUBTOTAL*		*315,145* *(21.59 %)*	*1562* *(0.11 %)*	*7045* *(0.48 %)*	*74.74*	*110.76*	*7.81*	*110.02*	*272.73*	*31.65*
Central-West	DF	32,600 (2.23 %)	556 (0.04 %)	1555 (0.11 %)	70.92	98.92	21.86	68.13	156.47	39.23
	GO	60,304 (4.13 %)	372 (0.03 %)	1470 (0.10 %)	96.15	185.48	17.69	116.28	413.98	110.88
	MS	47,011 (3.22 %)	433 (0.03 %)	1536 (0.11 %)	84.75	62.36	2.60	103.59	154.73	13.67
	MT	59,485 (4.08 %)	46 (0.00 %)	289 (0.02 %)	80.71	65.22	3.46	120.47	217.39	20.76
*SUBTOTAL*		*199,400* *(13.66 %)*	*1407* *(0.10 %)*	*4850* *(0.33 %)*	*84.73*	*109.45*	*13.40*	*19.59*	*0.36*	*0.28*
Southeast	ES	7184 (0.49 %)	18 (<0.001 %)	71 (<0.001 %)	178.87	555.56	42.25	199.47	555.56	84.51
	MG	180,520 (12.367 %)	1486 (0.10 %)	4170 (0.29 %)	80.32	144.01	16.55	118.94	339.84	63.55
	RJ	85,179 (5.83 %)	762 (0.05 %)	2459 (0.17 %)	149.57	371.39	51.65	146.83	427.82	127.29
	SP	189,156 (12.96 %)	2279 (0.16 %)	5799 (0.40 %)	155.47	230.36	26.04	229.83	561.65	82.60
*SUBTOTAL*		*462,039* *(31.65 %)*	*4545* *(0.31 %)*	*12,499* *(0.86 %)*	*125.38*	*227.06*	*28.00*	*94.75*	*78.82*	*78.01*
South	PR	46,239 (3.17 %)	1023 (0.07 %)	2926 (0.20 %)	90.90	151.52	14.70	97.73	287.39	49.90
	RS	161,316 (11.05 %)	2048 (0.14 %)	4188 (0.29 %)	96.62	201.66	24.36	67.81	293.95	47.04
	SC	81,839 (5.61 %)	2001 (0.14 %)	4155 (0.28 %)	189.47	332.83	58.72	159.59	604.20	130.45
*SUBTOTAL*		*289,394* *(19.83 %)*	*5072* *(0.35 %)*	*11,269* *(0.77 %)*	*121.96*	*243.30*	*34.52*	*98.55*	*415.02*	*78.53*
**TOTAL**		**1405,145** **(96.26 %)**	**14,079** **(0.96 %)**	**40,486** **(2.77 %)**	**105.65**	**196.32**	**22.87**	**128.03**	**384.76**	**64.89**

States: AC - Acre; AM - Amazonas; AP - Amapá; PA - Pará; RO - Rondônia; RR - Roraima; TO - Tocantins, AL - Alagoas; BA - Bahia; CE - Ceará; RJ - Rio de Janeiro; SP - São Paulo; PR - Paraná; RS - Rio Grande do Sul; SC - Santa Catarina; MA - Maranhão; PB - Paraíba; PE - Pernambuco; PI - Piauí; RN - Rio Grande do Norte; SE - Sergipe; DF - Distrito Federal; GO - Goiás; MS - Mato Grosso do Sul; MT - Mato Grosso; ES - Espírito Santo, MG - Minas Gerais.

## Discussion

The main findings showed that the individuals were primarily women of working age with a low level of education. A significant number of health resources were used, totaling 2,177,086. These included consultations with doctors and physical therapists, imaging, and consultations with specialists. Although referrals to physical therapy were not frequent, records of care by physical therapists without a prior referral were found. The use of health resources was heterogeneous across the Brazilian regions. Medical consultations per state ranged from <0.001 to 38.44/1000 people, while consultations with physical therapists ranged from <0.001 to 11.60/1000 people.

The demographic characteristics of the participants included in this study were similar to those in previous studies, with a female predominance and an age range of 32 to 66 years,^3^^,^^8^^,^^31^ and low education level. This aspect is relevant given the association between physically demanding occupations and the worst quality of life often linked to the presence of back pain complaints.^32^^,^^33^

The results showed that less than 1 % of participants were referred to a physical therapist (G2), which is supported by previous studies from Brazil.^22^^,^^34^ Imaging was prescribed to around 9 % of the individuals, and 12.9 % were referred to medical specialists. The Ministry of Health recommends referring patients to medical specialists after all treatment possibilities failed in PHC; however, we were not able to confirm this recommendation due to lack of detailed information. We might assume that a share of these individuals presented radiating pain or red flags, which may justify imaging and specialist care according to guidelines. However, because we did not have detailed information from the medical records within this system, this should be confirmed in future analytical studies.

The rate of referrals to physical therapists was somewhat below those reported in previous research.^6^^,^^8^^,^^35^, ^36^, ^37^ For instance, a previous study in the Netherlands showed a referral rate of around 16 % to physical therapists.^38^ Similarly, a study conducted in the United States showed that 16.3 % of patients received a referral for physical therapy within 90 days.^8^ Also, most consultations with physical therapists occurred without referral, although it is not clearly stated that this professional is a first line contact in the Brazilian Primary Care Policy. Nevertheless, our findings showed that individuals who consulted directly with physical therapists did not use a considerable amount of health resources, such as imaging and specialists' care. Approximately 4 % of the participants consulted a physical therapist (i.e., G2 and G3). This rate might be considered low compared to previous studies showing rates between 7 % and 16 %.^6^^,^^8^^,^^11^^,^^35^^,^^37^ Previous studies associated the low number of referrals with aspects ranging from problems with the administrative flow of referrals, communication barriers between levels of care, and insufficient number of physical therapists working in PHC. Another relevant aspect was the lack of understanding pertaining to the actual role of physical therapists working in PHC.^34^^,^^39^

More than 40,000 people accessed physical therapy without prior referral (i.e., G3 group), seeking care on their own, thus indicating possible challenges or limitations in accessing physical therapy services in PHC in Brazil. According to the Brazilian policy, physical therapists and other e-multi professionals are not considered a gateway to the health system. Patients need to be monitored; their health needs to be planned and evaluated together with family health teams.^19^^,^^35^ Nevertheless, these findings raise a novel discussion about the role of the physical therapist as a first contact professional, which is quite common in other countries^40^ but is not common in Brazil.

This study showed that, on average, about half of referrals to physical therapy were made 200 days after the initial consultation. However, this finding must be interpreted with caution, as delays in referral may be associated with new episodes of LBP unrelated to the initial medical consultation or even waiting lists. We observed a higher rate of health resource use in G2 compared to the other groups, mainly for imaging and specialist consultations. This may indicate that referrals to physical therapy may have occurred in addition to diagnostic procedures. These findings are similar to a previous study in Brazil, which showed a higher number of drug prescriptions and imaging for people with LBP, and a low rate of referral to physical therapy.^22^

### Study limitations

The present study has limitations inherent to research using secondary data, such as incomplete data. This could be explained by the incomplete implementation of the health system. Missing data, such as procedure codes, made it impossible to determine which actions were carried out, such as those carried out by nurses, which include medication administration and management. Missing data also limited the identification of the exact time of referral to physical therapists. The inclusion of non-specific ICD and CIAP codes also made it difficult to stratify individuals according to clinical conditions such as radiating pain.

## Conclusion

The present results showed a rather low referral of people with LBP to physical therapy in primary care in Brazil, and a delay between referral and first contact with this professional. In addition, imaging and specialist referrals were more common. About half of the people were referred to physical therapy after 200 days. The role of physical therapists as first-line professionals was not expected and warrants further studies to understand the potential benefits and necessary policy changes.

## Authors contributions

TLL, RLC: were involved in the conception and design; TLL, RLC: responsible for project administration and conduction of the study; TLL, PLM, HMP: data analyses and interpretation; TLL, RLC, PLM, LGM, RO, HMP: wrote the first draft of the manuscript, reviewed, and approved the final version of the manuscript. TLL had full access to all the data in the study and takes responsibility for the integrity of the data and the accuracy of the data analysis.

## Funding

The study was supported by the 10.13039/501100005668Research Support Foundation of the Federal District (FAPDF), protocol no. 00193-00000814/2021-21; Coordination for the 10.13039/501100002322Improvement of Higher Education Personnel (CAPES), funding code 001; PPGCR/UnB; UnB/DPI.

## Declaration of competing interest

The authors declare no competing interests.
